# Supplementary material for: IL-36 promotes anti-viral immunity by boosting sensitivity to IFN-α/β in IRF1 dependent and independent manners
Source: Nat Commun. 2019 Oct 16;10:4700. doi: 10.1038/s41467-019-12318-y (PMC6795910; doi:10.1038/s41467-019-12318-y)
Supplement: Supplementary file 1 — Supplementary Information [file 41467_2019_12318_MOESM1_ESM.pdf]

IL-36 promotes anti-viral immunity by boosting sensitivity to IFN-α/β in IRF1 dependent and independent manners

Wang et al.

Supplementary Table 1

Gene symbol	*p*-value	Fold change
Aim2	0.5401	1.06
Atg12	0.5253	1.12
Atg5	0.0485	0.62
Azi2	0.4455	1.24
Card9	0.0833	0.62
Casp1	0.7358	1.11
Casp8	0.1889	0.92
Ccl3	0.7973	0.94
Ccl4	0.0238	0.56
Ccl5	0.0935	0.62
Cd40	0.3033	0.81
Cd80	0.1587	1.04
Cd86	0.0255	1.14
Chuk	0.3204	0.64
Cnpy3	0.3209	0.88
Ctsb	0.3526	1.47
Ctsl	0.4159	1.43
Ctss	0.3746	0.15
Cxcl10	0.0118	0.44
Cxcl11	0.3671	0.73
Cxcl9	0.3908	0.74
Cyld	0.7822	0.86
Tkfc	0.6179	0.97
Ddx3x	0.3376	0.64
Ddx58	0.0010	0.36
Dhx58	0.0054	0.37
Fadd	0.0079	0.82
Fos	0.0011	0.72
Hsp90aa1	0.6029	1.13
Ifih1	0.4032	1.55
Ifna2	0.0686	0.59
Ifnar1	0.4621	0.54
Ifnb1	0.0067	0.63
Ikbkb	0.7663	0.81
Il12a	0.1587	1.13
Il12b	0.3564	0.6
Il15	0.2851	0.78
Il18	0.1319	0.87
Il1b	0.1190	0.79
Il6	0.0897	0.74
Irak1	0.0120	0.59
Irf3	0.1239	0.86

Gene symbol	*p*-value	Fold change
Irf5	0.0075	0.39
Irf7	0.0877	0.5
Isg15	0.6530	0.83
Jun	0.3513	1.36
Map2k1	0.1400	0.64
Map2k3	0.2076	0.84
Map3k1	0.6393	0.95
Map3k7	0.3969	1.59
Mapk1	0.5890	1.26
Mapk14	0.1402	0.67
Mapk3	0.5673	1.1
Mapk8	0.4058	0.74
Mavs	0.2353	1.16
Mefv	0.0911	0.73
Mx1	0.0322	0.46
Myd88	0.4317	1.39
Nfkb1	0.0769	0.55
Nfkbia	0.1697	0.9
Nlrp3	0.2261	1.31
Nod2	0.0001	0.28
Oas2	0.5494	1.16
Pin1	0.1586	1.23
Pstpip1	0.0093	0.43
Pycard	0.2812	0.74
Rela	0.4590	0.91
Ripk1	0.0032	0.34
Spp1	0.9287	0.93
Stat1	0.3380	0.71
Sugt1	0.4393	1.49
Tank	0.9287	1.07
Tbk1	0.8229	1.02
Tbkbp1	0.8610	1.02
Ticam1	0.2601	1.81
Tlr3	0.6145	0.85
Tlr7	0.2247	0.42
Tlr8	0.6082	0.81
Tlr9	0.0680	0.28
Tnf	0.5083	0.84
Tradd	0.0564	0.79
Traf3	0.0788	0.58
Traf6	0.3626	0.85
Trim25	0.3694	1.63

Supplementary Table 1: PCR array analyses of antiviral immune responses in wild type and IL-36β KO mice. Significantly decreased and increased mRNAs in IL-36β KO mice are shown in green and red, respectively (n = 3 per group). Source data are provided as a Source Data file.

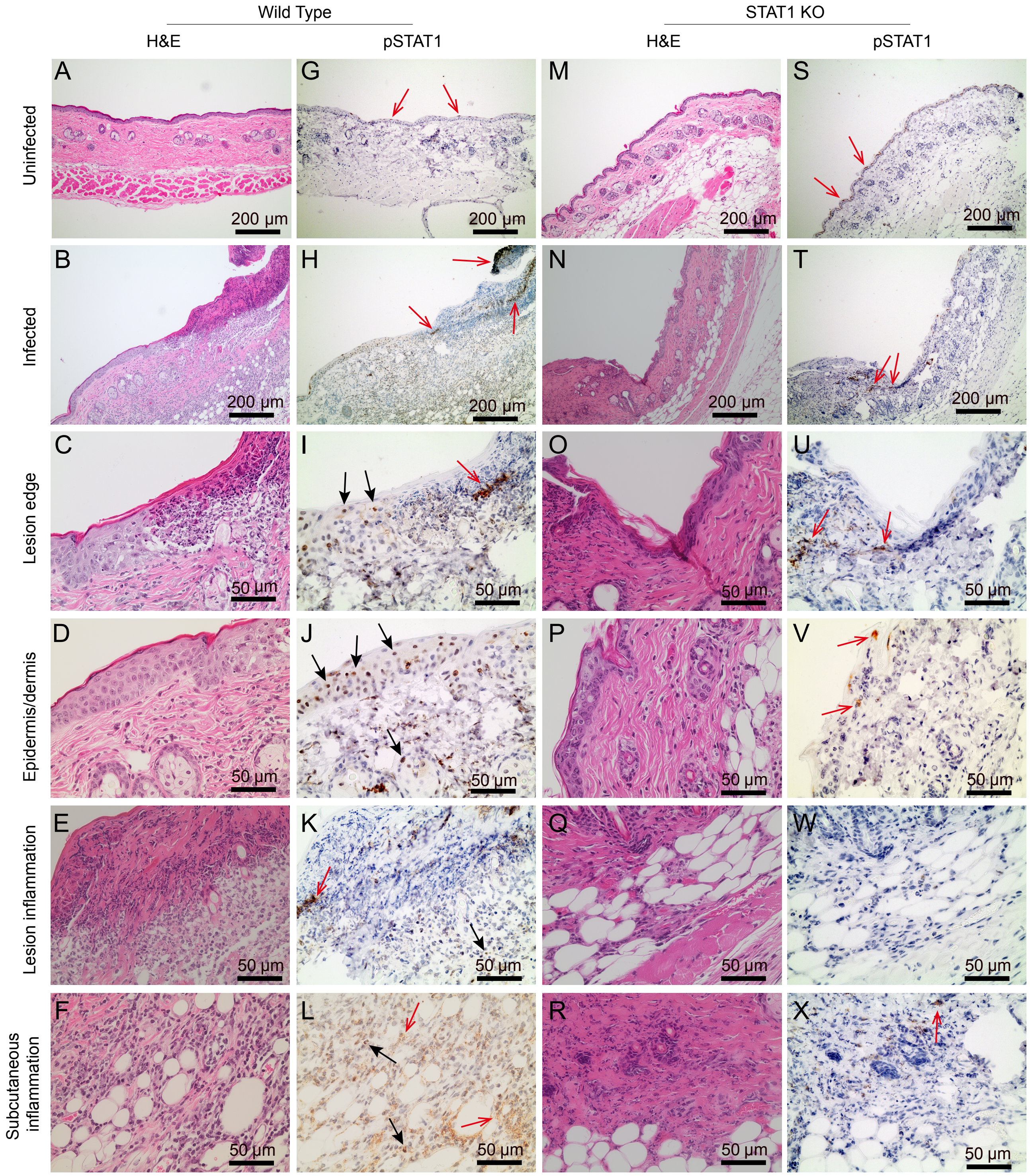

Supplementary Figure 1: STAT1 is activated in keratinocytes and immune cells recruited to lesions during HSV-1 skin infection. Wild type and STAT1 KO mice were infected with HSV-1 and primary infection sites collected 4 days later. Consecutive H&E and pSTAT1 stained skin sections are shown. Black and red arrows point to specific and non-specific staining, respectively.

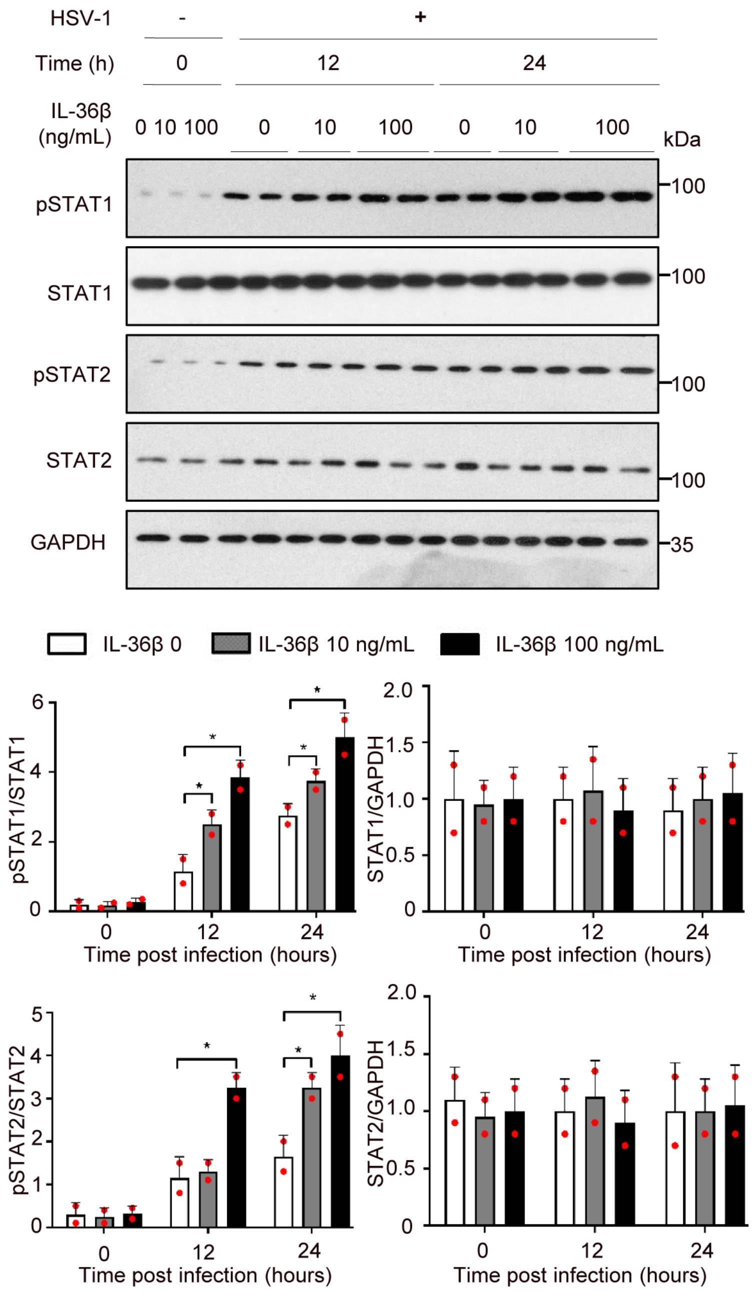

Supplementary Figure 2: IL-36β promotes enhanced STAT1 and STAT2 activation during HSV-1 infection of human keratinocytes. Human keratinocytes were stimulated with medium only or IL-36β for 12 h, followed by infection with 0.01 MOI HSV-1. Cell lysates were collected at the indicated time-points and pSTAT1, pSTAT2, STAT1, and STAT2 measured by immunoblotting and ImageJ analyses using GAPDH as the control. Quantitative data are shown as means ± SD. *, $p < 0.05$ (one-way ANOVA, n = 2 biologically independent samples per group, each red dot represents a single data point). Representative data from one of three independent experiments. Source data are provided as a Source Data file.

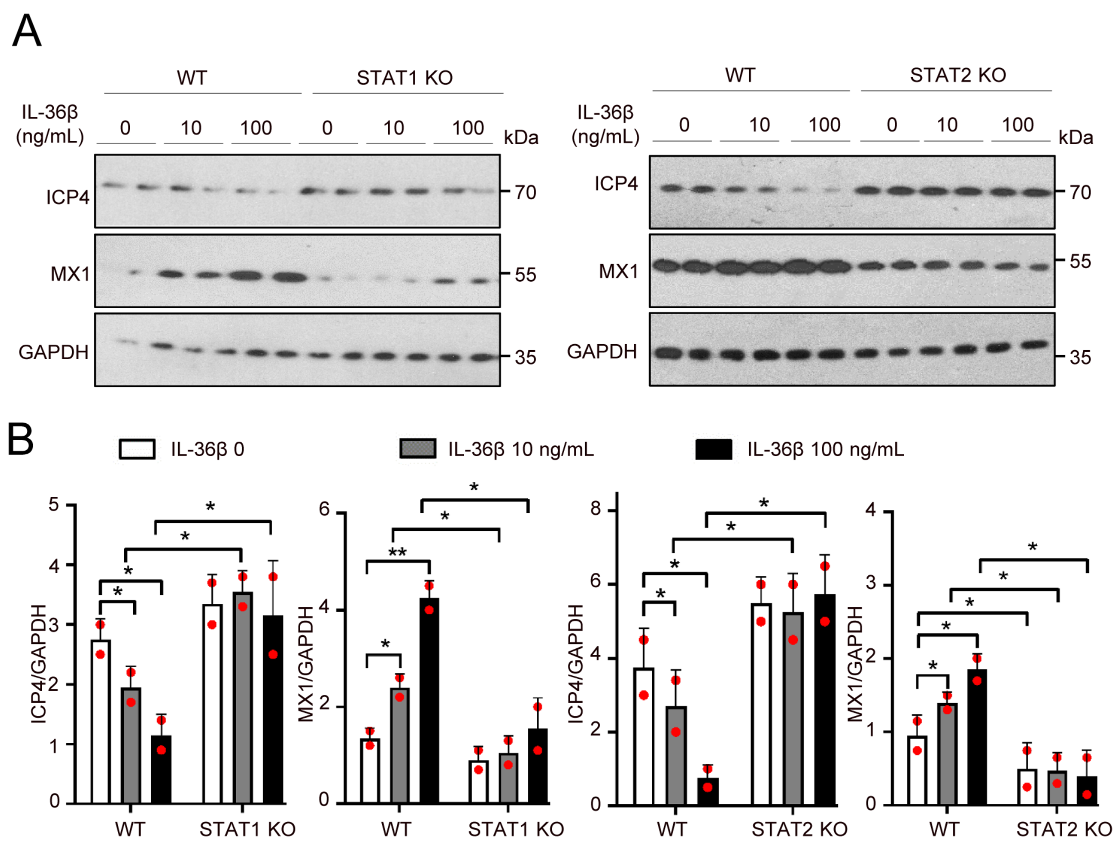

Supplementary Figure 3: IL-36β enhanced antiviral state and ISG expression is dependent upon STAT1 and STAT2 in female cells. (A) Wild type and *Stat1*$^{-/-}$ primary female mouse keratinocytes were treated with medium only or IL-36β followed by HSV-1 infection (MOI = 0.01) for 24 hours. Levels of HSV-1 ICP4 and host Mx1 were examined by Western blotting followed by ImageJ analyses. (B) Wild type and *Stat2*$^{-/-}$ primary female mouse keratinocytes were examined after IL-36β pre-treatment and HSV-1 infection using Western blotting and ImageJ analyses. (A-B) Quantitative data are shown as means ± SD (n = 2 biologically independent samples per group, each red dot represents a single data point). *, $p < 0.05$ (one-way ANOVA); **, $p < 0.01$. Source data are provided as a Source Data file.

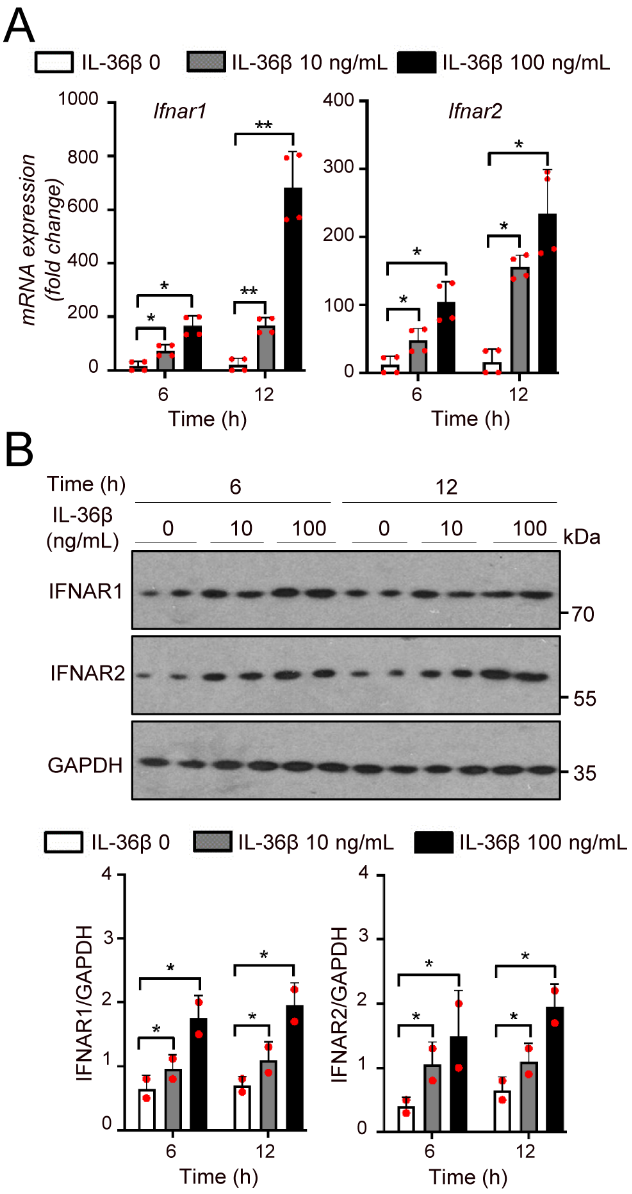

Supplementary Figure 4: IFNAR expression is induced by IL-36β in female mouse primary keratinocytes. (A) *Ifnar1* and *Ifnar2* mRNA expression was analyzed by real-time PCR in female mouse primary keratinocytes treated with medium only or IL-36β as indicated. (B) Mouse IFNAR1 and IFNAR2 protein expression was examined by Western blotting and ImageJ analyses in female cells. Quantitative data are shown as means ± SD (n = 2 biologically independent samples per group, each red dot represents a single data point). *, $p < 0.05$ (one-way ANOVA); **, $p < 0.01$. Source data are provided as a Source Data file.

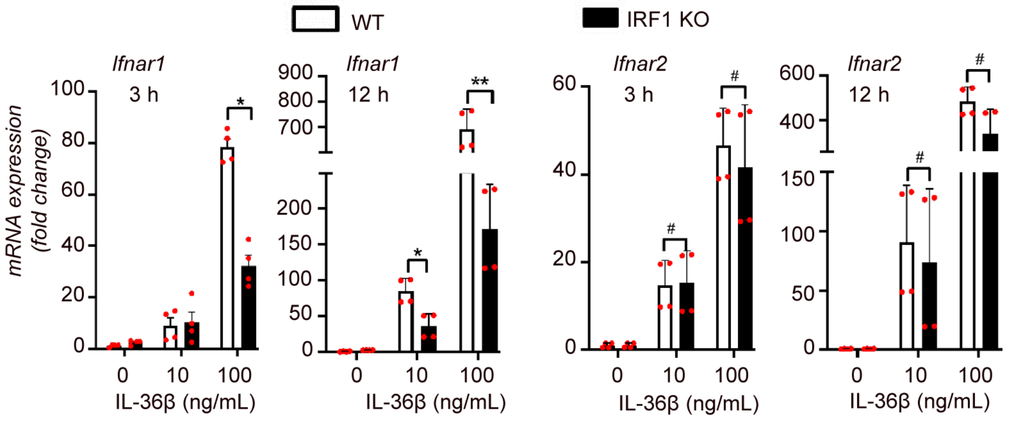

Supplementary Figure 5: IL-36β induced *Ifnar1* mRNA expression is partially dependent upon IRF1 in mouse cells. Expression levels of the *Ifnar1* and *Ifnar2* mRNAs were determined in mouse wild type and *Irf1* KO primary keratinocytes after treatment with medium only or IL-36β. Quantitative data are shown as means ± SD (n = 2 biologically independent samples per group, each red dot represents a single data point). *, $p < 0.05$ (one-way ANOVA); **, $p < 0.01$; #, $p > 0.05$. Source data are provided as a Source Data file.

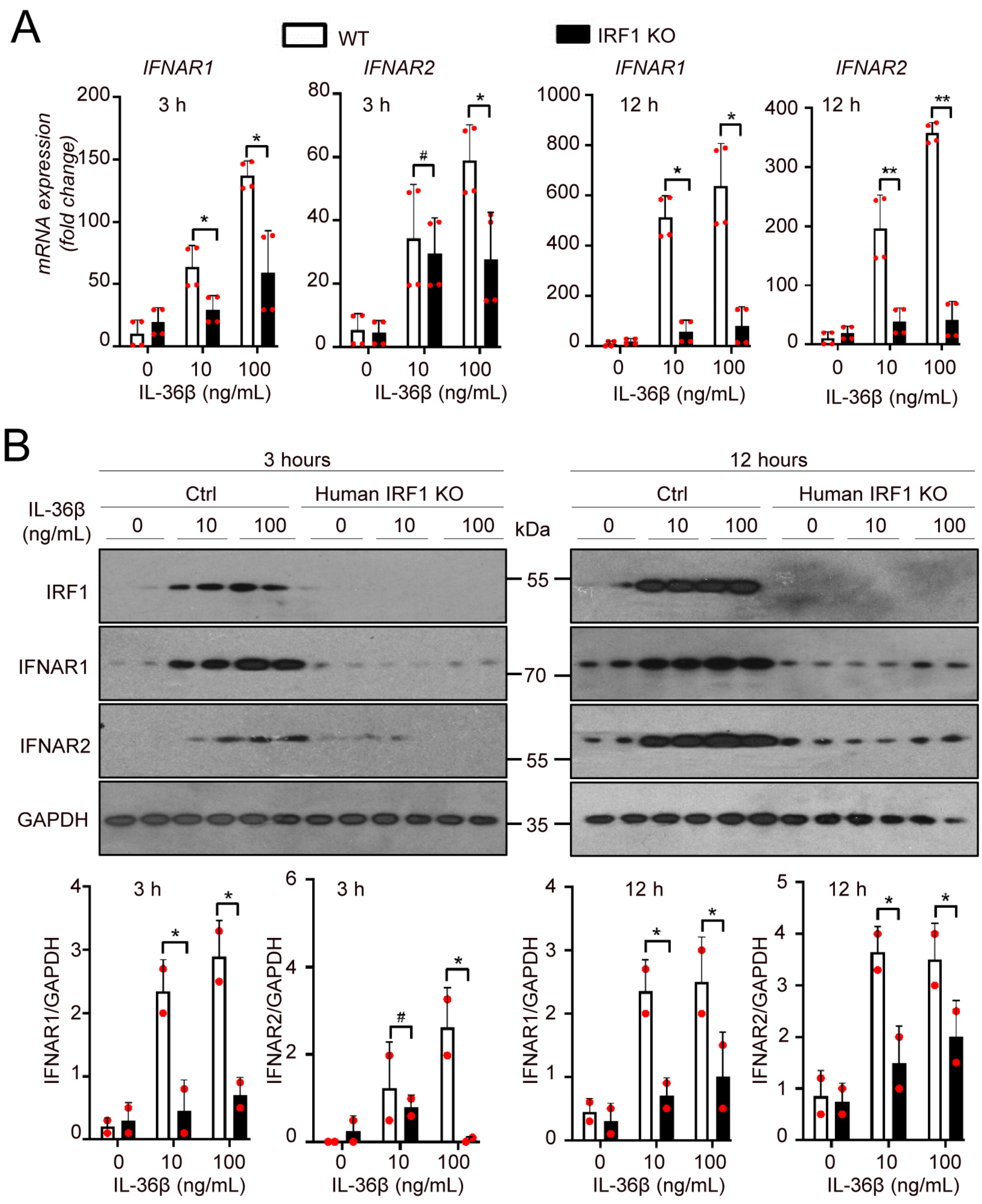

Supplementary Figure 6: IFNAR mRNA and protein expression is dependent upon IRF1 in human cells. (A) *IFNAR1* and *IFNAR2* mRNA expression in human control (Ctrl) and IRF1$^{-/-}$ keratinocytes was examined following medium only or IL-36β treatment at indicated time-points. (B) Protein levels of IFNAR1 and IFNAR2 in human control (Ctrl) and IRF1$^{-/-}$ keratinocytes were determined by Western blotting at indicated time-points. Quantitative data are shown as means ± SD (n = 2 biologically independent samples per group, each red dot represents a single data point). *, $p < 0.05$ (one-way ANOVA). Source data are provided as a Source Data file.

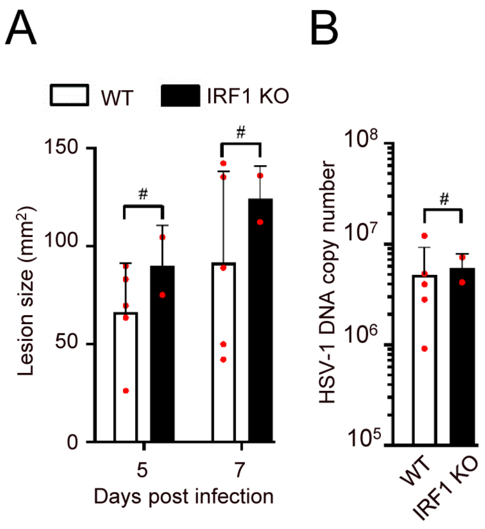

Supplementary Figure 7: Impact of IRF1 deficiency on HSV-1 skin infection in female mice. (A-B) Wild type and IRF1 KO female mice (WT: n = 5; KO: n = 2) were infected with HSV-1. #, p > 0.05. (A) Lesion sizes were measured. (B) HSV-1 DNA copy numbers in skin were determined 6 days after infection. Quantitative data are shown as means ± SD. #, p > 0.05 (one-way ANOVA). Each red dot represents a single mouse. Source data are provided as a Source Data file.

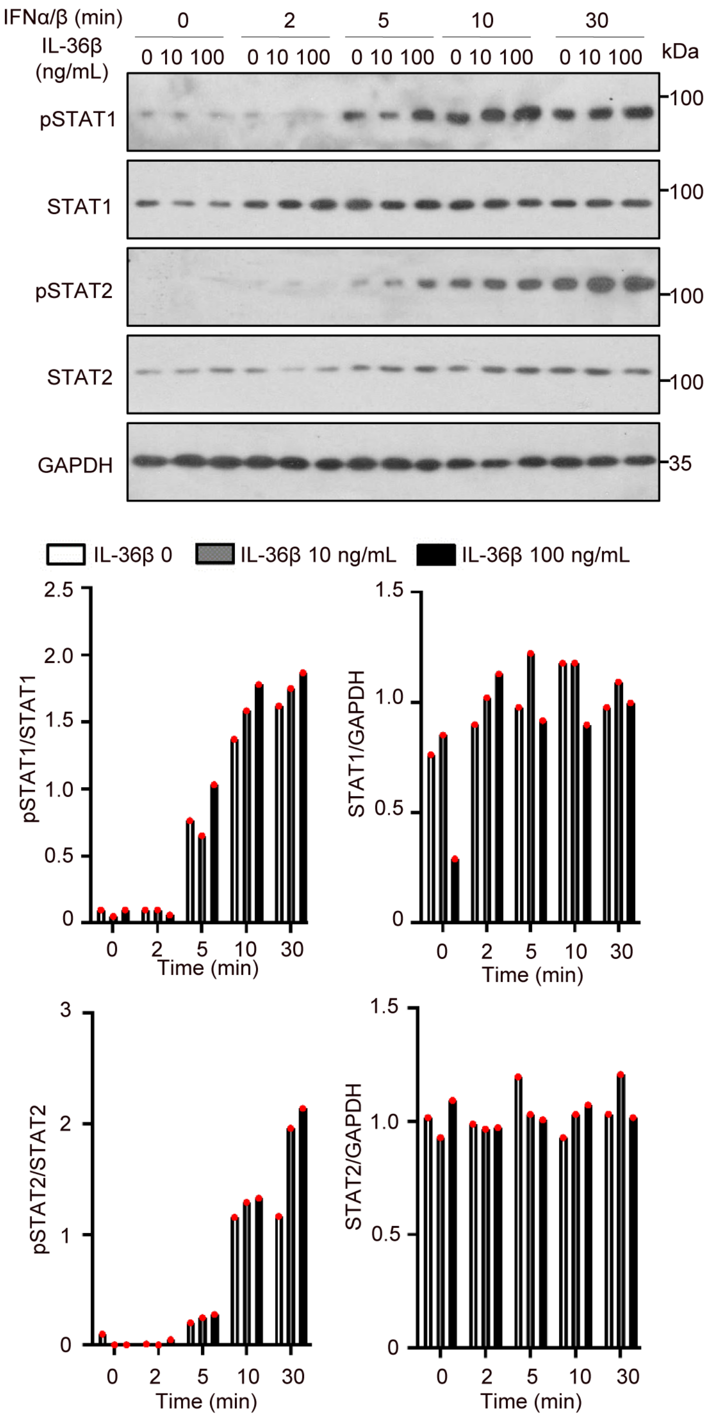

Supplementary Figure 8: STAT1/2 activation in the presence of high concentrations of type I IFN. Human keratinocytes were treated with medium only or IL-36β as indicated for 12 hours. Cells were subsequently treated with 0.1 ng/mL type I IFN-α/β and protein analyzed by Western blotting at the indicated time points (n = 1 biologically independent sample per group, each red dot represents a single data point). Source data are provided as a Source Data file.

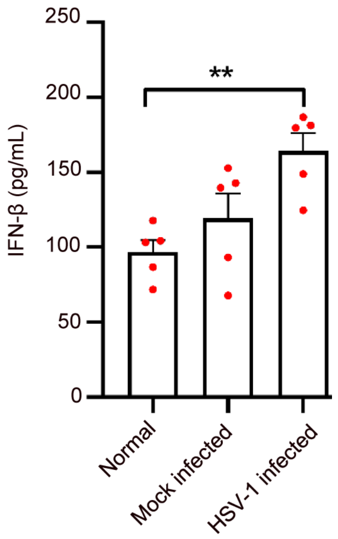

Supplementary Figure 9: IFN-β is secreted at increased levels from HSV-1 infected skin. IFNAR1 KO mice (n = 5 per group) were left untreated, mock infected or infected with HSV-1. Skin was collected using 8 mm punch biopsy tools after 2 days, and the skin placed in 200 μL keratinocyte culture medium for 24 hours. Levels of IFN-β were determined by ELISA. **, $p < 0.01$ (one-way ANOVA). Quantitative data are shown as means ± SD (each red dot represents a single data point). Note: the skin is wounded to collect the samples for explant culture. Source data are provided as a Source Data file.

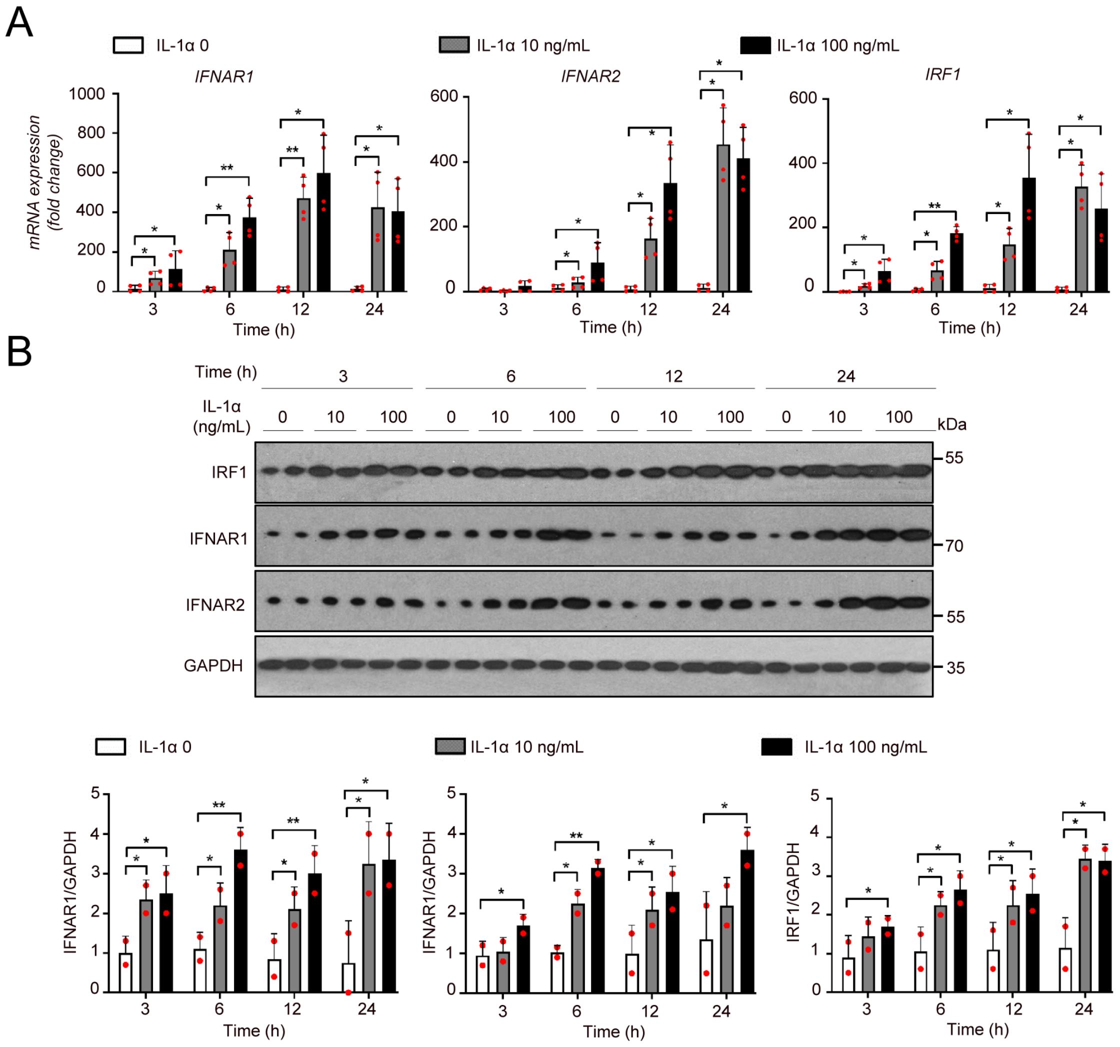

Supplementary Figure 10: IL-1 induces expression of IFNAR1, IFNAR2 and IRF1 in human keratinocytes. Human keratinocytes were treated with medium only or IL-1α as indicated. Expression of IFNAR1, IFNAR2 and IRF1 was examined by real-time PCR (A) and Western blotting (B). Quantitative data are shown as means ± SD (n = 2 biologically independent samples per group, each red dot represents a single data point). *, $p < 0.05$ (one-way ANOVA); **, $p < 0.01$. Source data are provided as a Source Data file.

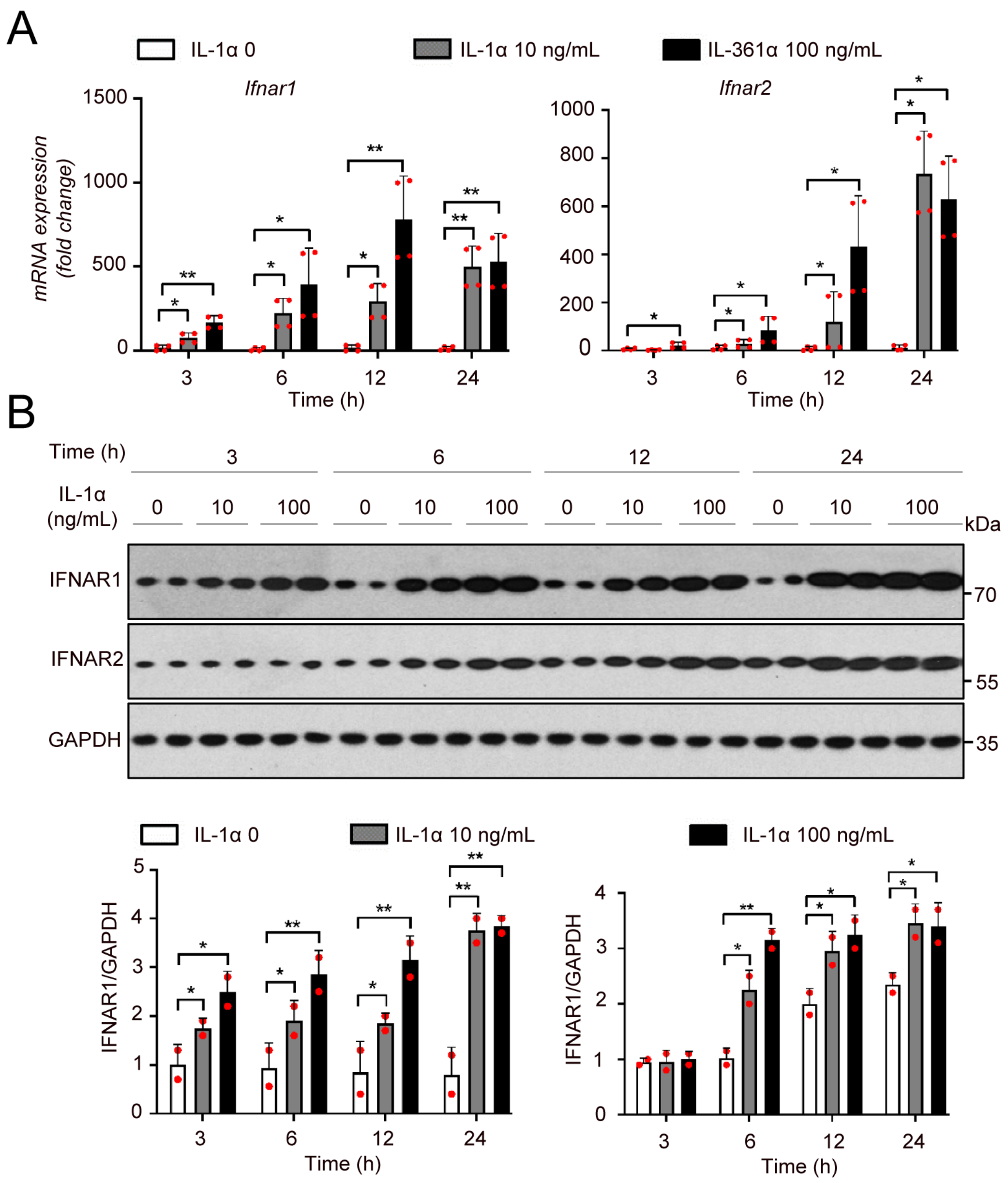

Supplementary Figure 11: IL-1 increases expression of the type I IFN receptor in mouse keratinocytes. Mouse keratinocytes were treated with medium only or IL-1α and expression of IFNAR1 and IFNAR2 mRNA (A) and protein (B) examined by real-time PCR and Western blotting, respectively. Quantitative data are shown as means ± SD (n = 2 biologically independent samples per group, each red dot represents a single data point). *, $p < 0.05$ (one-way ANOVA); **, $p < 0.01$. Source data are provided as a Source Data file.

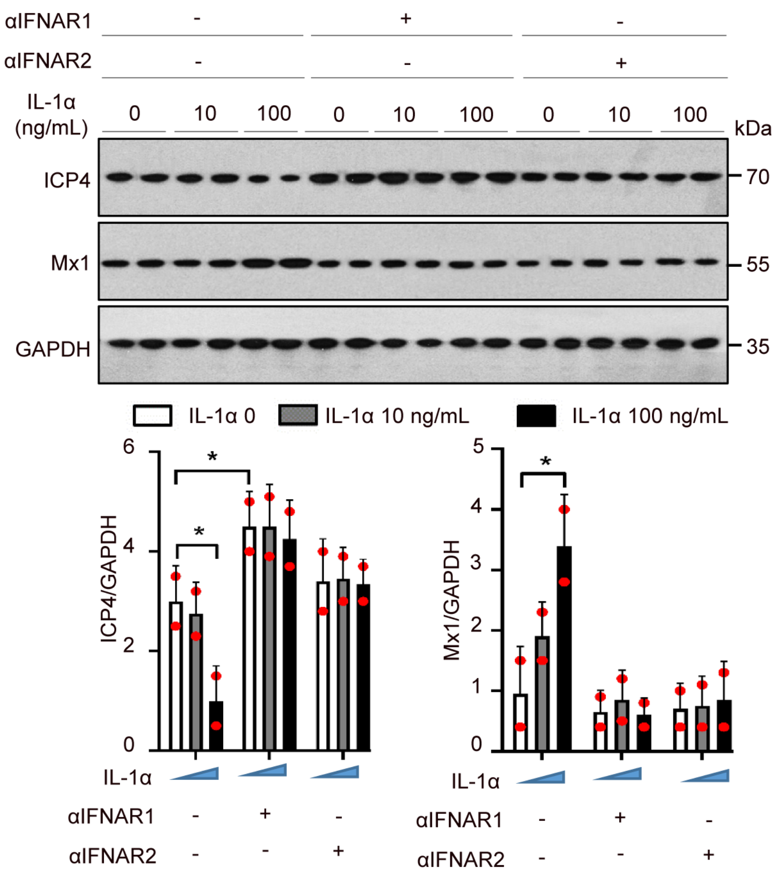

Supplementary Figure 12: Anti-viral response induced by IL-1 in mouse keratinocytes is dependent upon IFNAR1 and IFNAR2. Mouse keratinocytes were pre-treated with medium only or IL-1α. Cells were infected with HSV-1 in the presence of neutralizing antibodies against IFNAR1 or IFNAR2 or isotype matched antibodies. Levels of ICP4 and Mx1 were evaluated by Western blotting. Quantitative data are shown as means ± SD (n = 2 biologically independent samples per group, each red dot represents a single data point). *, $p < 0.05$ (one-way ANOVA). Source data are provided as a Source Data file.

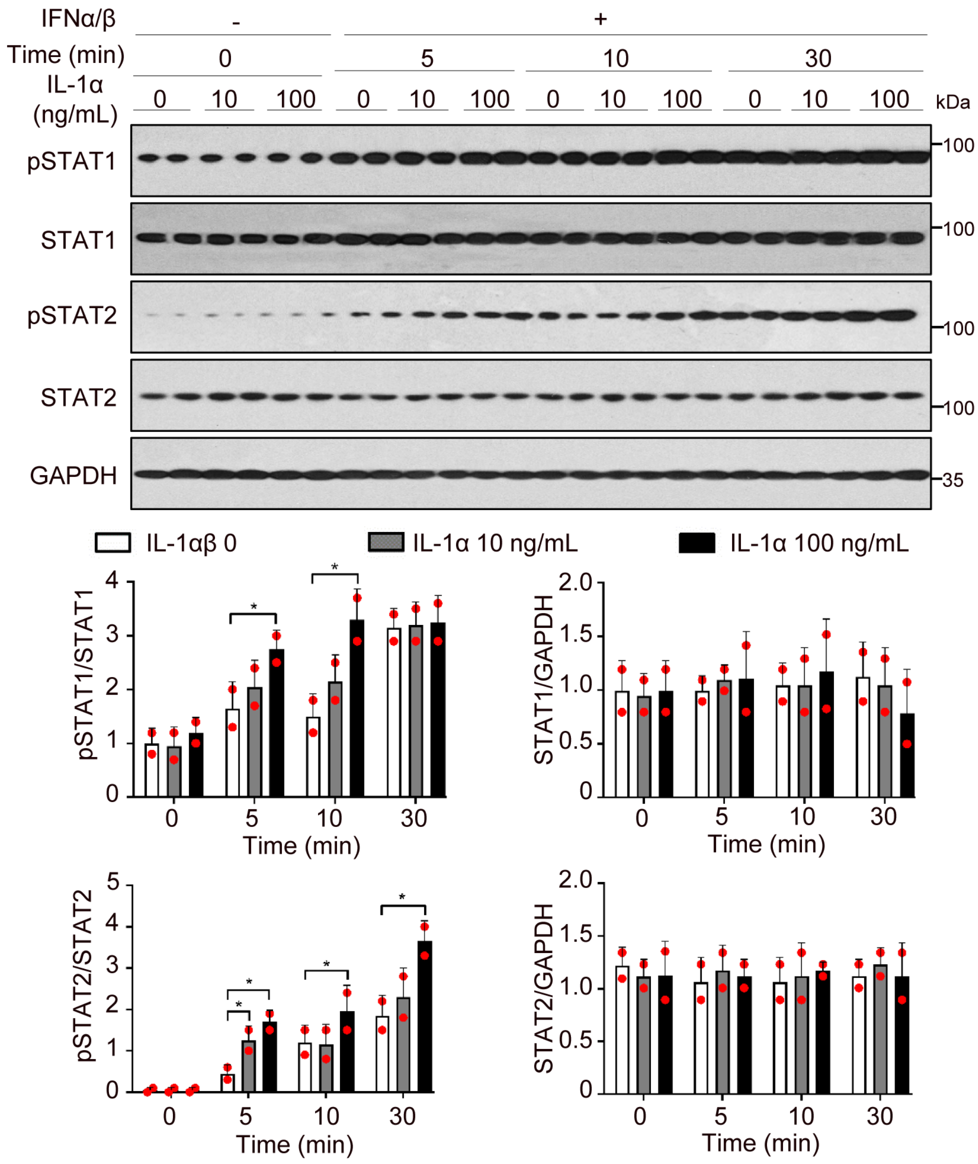

Supplementary Figure 13: IL-1 enhances STAT1 activation in response to type I IFN. Mouse keratinocytes were pre-treated with IL-1α before addition of IFN-α/β. Levels of pSTAT1, STAT1, pSTAT2, and STAT2 were determined by Western blotting. Quantitative data are shown as means ± SD (n = 2 biologically independent samples per group, each red dot represents a single data point). *, $p < 0.05$ (one-way ANOVA). Source data are provided as a Source Data file.

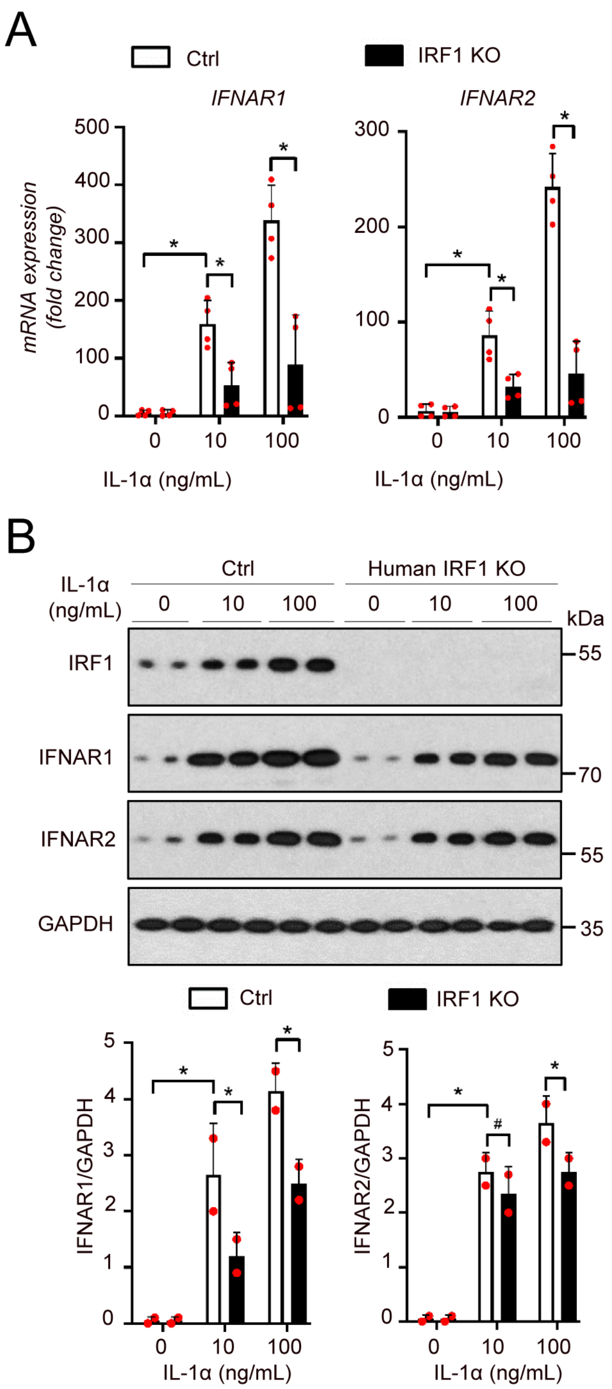

Supplementary Figure 14: IL-1 induced IFNAR expression is partially dependent upon IRF1. *IRF1* was edited using CRISPR/Cas9 in human keratinocytes. Cells were treated with medium only or IL-1α, and IFNAR1 and IFNAR2 expression examined by real-time PCR (A) and Western blotting. Quantitative data are shown as means ± SD (n = 2 biologically independent samples per group, each red dot represents a single data point). *, $p < 0.05$ (one-way ANOVA); **, $p < 0.01$. Source data are provided as a Source Data file.

GENE	PRIMER NAME	PRIMER SEQUENCE
STAT1	humanSTAT1-UP	TGGCAGACCCCAGACCGA
	humanSTAT1-DN	CGAGGATGGCATACAGCAAAT
STAT2	humanSTAT2-UP	GGGGACTGCAACCCTAATC
	humanSTAT2-DN	TTCAATCCAGACAGCCAAGTA
IFNAR1	humanIFNAR1-UP	TGACGCTGTATGTGAGAA
	humanIFNAR1-DN	TAAATGACAAACGGGAGA
IFNAR2	humanIFNAR2-UP	AGCGTCGGGTCCCAGAGC
	humanIFNAR2-DN	GAAGGCATTCTGGCTCAAAA
IRF1	humanIRF1-UP	TCCGGAGCTGGGCCATTCAC
	humanIRF1-DN	GCTGAGCTGCCCTTGTTCC
HSV1 gD	HSV-1 gD-F	CTACTATGACAGCTTCAGCG
	HSV-1 gD-R	CCGTCCAGTCGTTTATCTTC
HSV1 ICP4	HSV-1 ICP4-F	GCCGGGACTCTTGCGCTTGC
	HSV-1 ICP4-R	CGGACCTGCTGTTTGAGAACCA
Stat1	mouseSTAT1-UP	CGCCCCTCAGACCCACTT
	mouseSTAT1-DN	GGTATGGAGCAGAGCTGAAAC
Stat2	mouseSTAT2-UP	TTCCGCTGTTCGCTATCTT
	mouseSTAT2-DN	ATCTCCCACTGCGCCATT
Ifnar1	mouseIFNAR1-UP	CAAGACGATGCTCGCTGTC
	mouseIFNAR1-DN	TTCTCAGGAGGTTTCAGATTTT
Ifnar2	mouseIFNAR2-UP	CCCAGGACATCCCAGAGG
	mouseIFNAR2-DN	GAGGAGACCGACGGCAGA
Irf1	mouseIRF1-UP	CAGAGGGACCCCAGCATCTC
	mouseIRF1-DN	TTTTGTTCCACGGCACCCG
Mx1	mouseMX1-UP	GGTGCTGAAATTGAGGAA
	mouseMX1-DN	GAAGTTCTGACCCTTGTTG
Irf5	mouseIRF5-UP	TCTGTAGAGGGTCGCTCCG
	mouseIRF5-DN	AGTGGTTCATGGCAAAGG
Ifnb1	mouseIFN-β-UP	GGTGCTGAAATTGAGGAA
	mouseIFN-β-DN	GAAGTTCTGACCCTTGTTG
Fadd	mouseFADD-UP	CGCCGACACGATCTACTG
	mFADD-DN	ACAATGTCAAATGCCACC
Nod2	mouseNOD2-UP	GGGGAAGAGGGTGGTTCA
	mouseNOD2-DN	TCCTGCGAGCACATTTCA
Cxcl4	mouseCXCL4-UP	GGGGAAGAGGGTGGTTCA
	mCXCL4-DN	TCCTGCGAGCACATTTCA
Oas1	mouseOAS1-UP	CTGCTGAAGGAGGTGAAG
	mouseOAS1-DN	ACTGATGAGATTGGCGTAG
Eif2ak2	mouseEIF2AK2-UP	AGACATCCTTCGTGCTGC
	mouseEIF2AK2-DN	TTGCCCAAGTATTCACCC
Isg15	mouseISG15-UP	GACCTAGAGCTAGAGCCTG
	mouseISG15-DN	TCATGGAGTTAGTCACGG
Ifitm2	mouseIFITM2-UP	CCATGTGGTCTGGTCCCT
	mouseIFITM2-DN	CCACCATCTTCCTGTCCC
Ifitm3	mouseIFITM3-UP	GTCCCTGTTCAATACACTCTT
	mouseIFITM3-DN	CACATCACCCACCATCTT
Ifit3	mouseIFIT3-UP	CAGCAGCACAGAAACAGA
	mouseIFIT3-DN	AATTCCAGGTGAAATGGC

Supplementary Table 2: Primers used for real-time PCR.
