# Supplementary material for: IL-36 promotes anti-viral immunity by boosting sensitivity to IFN-α/β in IRF1 dependent and independent manners
Source: Nat Commun. 2019 Oct 16;10:4700. doi: 10.1038/s41467-019-12318-y (PMC6795910; doi:10.1038/s41467-019-12318-y)
Supplement: Supplementary file 3 — Reporting Summary [file 41467_2019_12318_MOESM3_ESM.pdf]

nature research

Corresponding author(s): Liselotte Jensen
Last updated by author(s): Aug 20, 2019

Reporting Summary

Nature Research wishes to improve the reproducibility of the work that we publish. This form provides structure for consistency and transparency in reporting. For further information on Nature Research policies, see Authors & Referees and the Editorial Policy Checklist.

Statistics

For all statistical analyses, confirm that the following items are present in the figure legend, table legend, main text, or Methods section.

n/a	Confirmed	
☐	☒	The exact sample size (*n*) for each experimental group/condition, given as a discrete number and unit of measurement
☐	☒	A statement on whether measurements were taken from distinct samples or whether the same sample was measured repeatedly
☐	☒	The statistical test(s) used AND whether they are one- or two-sided<br>*Only common tests should be described solely by name; describe more complex techniques in the Methods section.*
☒	☐	A description of all covariates tested
☐	☒	A description of any assumptions or corrections, such as tests of normality and adjustment for multiple comparisons
☐	☒	A full description of the statistical parameters including central tendency (e.g. means) or other basic estimates (e.g. regression coefficient) AND variation (e.g. standard deviation) or associated estimates of uncertainty (e.g. confidence intervals)
☐	☒	For null hypothesis testing, the test statistic (e.g. *F*, *t*, *r*) with confidence intervals, effect sizes, degrees of freedom and *P* value noted<br>*Give P values as exact values whenever suitable.*
☒	☐	For Bayesian analysis, information on the choice of priors and Markov chain Monte Carlo settings
☒	☐	For hierarchical and complex designs, identification of the appropriate level for tests and full reporting of outcomes
☒	☐	Estimates of effect sizes (e.g. Cohen's *d*, Pearson's *r*), indicating how they were calculated

Our web collection on statistics for biologists contains articles on many of the points above.

Software and code

Policy information about availability of computer code

Data collection	Image J was used for analyses of Western blot films. The software is freely available from https://imagej.nih.gov/ij/. The link is provided in the methods section.
Data analysis	Image J was used for analyses of Western blot films. The software is freely available from https://imagej.nih.gov/ij/

For manuscripts utilizing custom algorithms or software that are central to the research but not yet described in published literature, software must be made available to editors/reviewers. We strongly encourage code deposition in a community repository (e.g. GitHub). See the Nature Research guidelines for submitting code & software for further information.

Data

Policy information about availability of data

All manuscripts must include a data availability statement. This statement should provide the following information, where applicable:

- Accession codes, unique identifiers, or web links for publicly available datasets
- A list of figures that have associated raw data
- A description of any restrictions on data availability

All data generated and analyzed during this study are included in this published article and its supplementary information and source data files

Field-specific reporting

Please select the one below that is the best fit for your research. If you are not sure, read the appropriate sections before making your selection.

☒ Life sciences ☐ Behavioural & social sciences ☐ Ecological, evolutionary & environmental sciences

Life sciences study design

All studies must disclose on these points even when the disclosure is negative.

Sample size	Sample sized was based on our previous experience with the model system used. No statistical methods were used for determining sample sizes.
Data exclusions	No data was excluded
Replication	Experiments were performed at least 3 independent times with several biological replicates in each experiment. Experiments were reproducible, except in one case involving IRF1 -/- cells, which is described in the manuscript; Results section 'The role of IRF1 in antiviral immunity is species dependent'.
Randomization	All mice available to us were used. The study did not involve allocation into separate experimental groups as we cannot influence genotypes that are by nature predetermined.
Blinding	Investigators were generally not blinded. All parameters examined were quantifiable; hence, no risk of subconscious bias.

Reporting for specific materials, systems and methods

We require information from authors about some types of materials, experimental systems and methods used in many studies. Here, indicate whether each material, system or method listed is relevant to your study. If you are not sure if a list item applies to your research, read the appropriate section before selecting a response.

Materials & experimental systems

n/a	Involved in the study	
☐	☒	Antibodies
☐	☒	Eukaryotic cell lines
☒	☐	Palaeontology
☐	☒	Animals and other organisms
☒	☐	Human research participants
☒	☐	Clinical data

Methods

n/a	Involved in the study	
☒	☐	ChIP-seq
☒	☐	Flow cytometry
☒	☐	MRI-based neuroimaging

Antibodies

Antibodies used	Mouse monoclonal antibodies to HSV-1 ICP4 (H943, Catalog # sc-69809, RRID:AB_844234, used at 1:500 dilution), IFN-α/βRα Antibody (H-11, Catalog # sc-7391, RRID:AB_2122749, used at 1:800 dilution), IFN-α/βRβ Antibody (F-7, Catalog # sc-137209, RRID:AB_2122750, used at 1:500 dilution) and Mx1 Antibody (E-8, Catalog # sc-398564, RRID:AB_1146318, used at 1:600 dilution) were acquired from Santa Cruz Biotechnology. Rabbit monoclonal antibodies to phospho-STAT1 (Tyr701, 58D6, Catalog # 9167, RRID: AB_561284, used at 1:800 dilution (Westerns) and 1:50 dilution (immunohistochemistry)), phospho-STAT2 (Tyr690, D3P2P, Catalog # 88410, RRID: AB_2800123, used at 1:800 dilution), STAT1 (D1K9Y, Catalog # 14994, RRID:AB_2799965, used at 1:1000 dilution (Westerns)), STAT2 (D9J7L, Catalog # 72604, RRID:AB_2799824, used at 1:1000 dilution), Gapdh (14C10, Catalog # 2118, RRID: AB_561053, used at 1:2000 dilution), IRF1 (D5E4, Catalog # 8478, RRID:AB_10949108, used at 1:1000 dilution), anti-rabbit IgG HRP-linked (Catalog # 7074, RRID: AB_2099233) or anti-mouse IgG HRP-linked (Catalog # 7076, RRID: AB_330924) antibodies (used at 1:10,000 dilution) were obtained from Cell Signaling Technology.
Validation	We extensively used knockout cells/mice and uninfected cells/mice as controls for mammalian and HSV-1 protein specific antibodies as described in the paper

Eukaryotic cell lines

Policy information about cell lines

Cell line source(s)	HaCaT cells were obtained from Meenhard Herlyn at Wistar Institute in Philadelphia, PA.
Authentication	We previously confirmed the HaCaT cell line through analyses of K10 and K14 expression
Mycoplasma contamination	The HaCaT cells were previously confirmed mycoplasma free; they were not specifically tested during this study but are not expanded more than max 20 passages before returning to the mycoplasma free stock
Commonly misidentified lines (See ICLAC register)	N/A

Animals and other organisms

Policy information about studies involving animals; ARRIVE guidelines recommended for reporting animal research

Laboratory animals	Male and female mice were used at approx 8 weeks of age and analyzed separately. C57BL/6J, Irf1−/−, Il36b -/-, Stat1-/-, Stat2-/- and Ifnar1−/−
Wild animals	No wild animals were used
Field-collected samples	N/A
Ethics oversight	Temple University Institutional Animal Care and Use Committee approved the procedures and perform regular inspections

Note that full information on the approval of the study protocol must also be provided in the manuscript.
